# FOXO protects against age‐progressive axonal degeneration

**DOI:** 10.1111/acel.12701

**Published:** 2017-11-26

**Authors:** Inah Hwang, Hwanhee Oh, Evan Santo, Do‐Yeon Kim, John W. Chen, Roderick T. Bronson, Jason W. Locasale, Yoonmi Na, Jaclyn Lee, Stewart Reed, Miklos Toth, Wai H. Yu, Florian L. Muller, Jihye Paik

**Affiliations:** ^1^ Department of Pathology and Laboratory Medicine Weill Cornell Medicine New York NY USA; ^2^ Department of Pharmacology School of Dentistry Kyungpook National University Daegu Korea; ^3^ Center for Systems Biology and the Division of Neuroradiology Department of Radiology Massachusetts General Hospital Harvard Medical School Boston MA USA; ^4^ Department of Microbiology and Immunobiology Harvard Medical School Boston MA USA; ^5^ Department of Pharmacology and Cancer Biology Duke University School of Medicine Durham NC USA; ^6^ Department of Medical Oncology Dana Farber Cancer Institute Boston MA USA; ^7^ Department of Pharmacology Weill Cornell Medicine New York NY USA; ^8^ Department of Pathology and Cell Biology Columbia University New York NY USA; ^9^ Cancer Systems Imaging The University of Texas MD Anderson Cancer Center Houston TX USA

**Keywords:** accelerated aging, aging, central nervous system, FOXO, mouse models, neurodegeneration, neuroinflammation, oxidative stress

## Abstract

Neurodegeneration resulting in cognitive and motor impairment is an inevitable consequence of aging. Little is known about the genetic regulation of this process despite its overriding importance in normal aging. Here, we identify the Forkhead Box O (FOXO) transcription factor 1, 3, and 4 isoforms as a guardian of neuronal integrity by inhibiting age‐progressive axonal degeneration in mammals. FOXO expression progressively increased in aging human and mouse brains. The nervous system‐specific deletion of *Foxo* transcription factors in mice accelerates aging‐related axonal tract degeneration, which is followed by motor dysfunction. This accelerated neurodegeneration is accompanied by levels of white matter astrogliosis and microgliosis in middle‐aged *Foxo* knockout mice that are typically only observed in very old wild‐type mice and other aged mammals, including humans. Mechanistically, axonal degeneration in nerve‐specific *Foxo* knockout mice is associated with elevated mTORC1 activity and accompanying proteotoxic stress due to decreased Sestrin3 expression. Inhibition of mTORC1 by rapamycin treatment mimics FOXO action and prevented axonal degeneration in *Foxo* knockout mice with accelerated nervous system aging. Defining this central role for FOXO in neuroprotection during mammalian aging offers an invaluable window into the aging process itself.

## 1 INTRODUCTION

The genetic regulation of aging in the nervous system and aging‐related neurodegenerative diseases remains one of the least understood aspects of mammalian biology. In particular, axonal tract degeneration is a common feature of neurodegenerative conditions and aging‐associated nervous system deterioration. Injury of the brain or the spinal cord, inflammatory disorders, and age‐related degenerative conditions all share “axonopathy” despite varying etiologies. However, the mechanism underlying the aging‐related axonopathy remains obscure, with important consequences for the treatment of devastating diseases such as Alzheimer's and Parkinson's disease and amyotrophic lateral sclerosis.

Extensive evidence in invertebrate model organisms highlighted the role of the FOXO transcription factor (DAF‐16 in *Caenorhabditis elegans*) in nervous system functions. DAF‐16 regulates learning and memory (Kauffman, Ashraf, Corces‐Zimmerman, Landis & Murphy, 2010; Murakami, Bessinger, Hellmann & Murakami, 2005), axonal degeneration and regeneration, and neurite outgrowth (Christensen, de la Torre‐Ubieta, Bonni & Colon‐Ramos, 2011). The hyperactivation of DAF‐16 in worm neurons is required for lifespan extension following loss of insulin‐like signaling (Wolkow, Kimura, Lee & Ruvkun, 2000). This genetic study points to the potential importance of FOXO in the nervous system as a central regulator of longevity.

Previous studies reported FOXO's role in both neuroprotection and neurodegeneration. The activation of the pro‐survival phosphoinositide‐3 kinase (PI3K)–AKT pathway protected against neuronal cell death in cases of nerve growth factor withdrawal, while the activation of FOXO in this context promoted cell death (Simon et al., 2016). Neuroprotective NMDA receptor signaling by PI3K–AKT activation inhibited FOXO‐dependent gene expression to suppress oxidative stress in neurons (Papadia et al., 2008). Similarly, activation of FOXO in mammalian neurons induced cell death (Gilley, Coffer & Ham, 2003; Yuan et al., 2008, 2009). By contrast, constitutively active FOXO protected neurons against excitotoxic and proteotoxic insults (Mojsilovic‐Petrovic et al., 2009).

Mechanistic target of rapamycin (mTOR) is a genetically and pharmacologically proven regulator of aging and aging‐related diseases (Johnson, Rabinovitch & Kaeberlein, 2013). It is a master activator of cellular growth processes including protein and lipid biosynthesis and a repressor of catabolic autophagy. Altered mTORC1 signaling is associated with neurological and neurodevelopmental disorders, such as epilepsy, microcephaly, and autism. Aberrant activation of mTORC1 and its normalization by rapamycin have been reported in studies on aging‐associated conditions and diseases such as Alzheimer's (Caccamo, Majumder, Richardson, Strong & Oddo, 2010; Spilman et al., 2010) and mouse models of laminopathy (Ramos et al., 2012). These studies emphasized increased proteotoxicity due to mTORC1 activation and insufficient autophagy as a disease driving mechanism.

Here, we investigated the role of FOXO in mammalian central nervous system aging both genetically and functionally. We hypothesized that functionally redundant and developmentally dispensable FOXO 1, 3, and 4 isoforms are necessary for healthy brain aging. To investigate the role of FOXO 1, 3, and 4 transcription factors together as broadly as possible in aging nervous system, we analyzed mice with combined deletion of these FOXO genes in both neurons and glia. We also tested whether loss of FOXO function in mature neurons is sufficient to initiate aging‐associated axonopathy. Functional study on elevated mTORC1 in FOXO 1, 3, and 4 null brains was performed to further prove their counteracting function in aging brain. Our study broadly defines the neuroprotective role for FOXO during aging and provides new insights into the function of FOXO in the nervous system.

## 2 RESULTS

### 2.1 Expression of FOXO increases in aging brain

**Figure 1:**
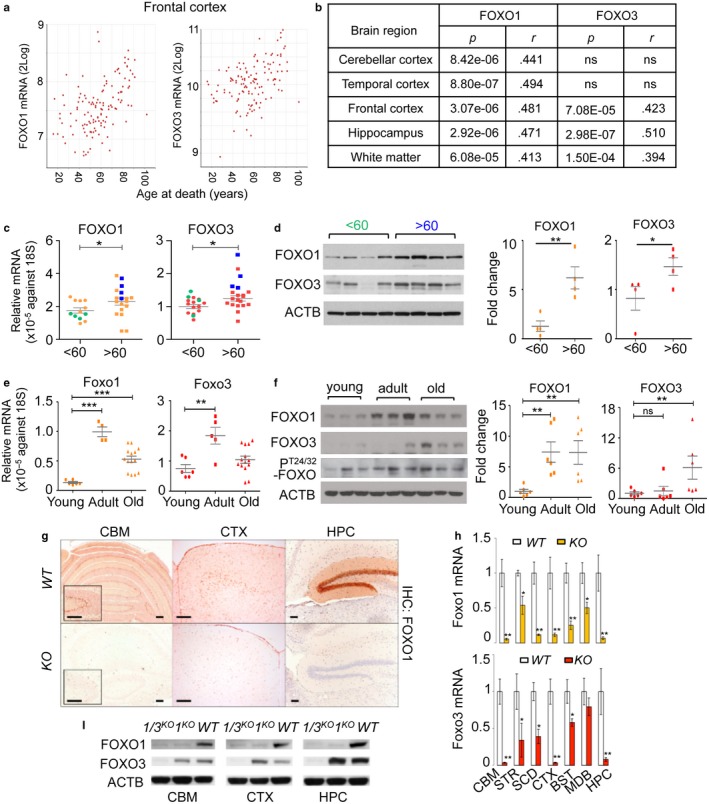
Expression of FOXO increases in aging brain. (a) XY plots of FOXO1 or FOXO3 mRNA expression within the noted brain regions vs. age of the subjects at time of death. (b) Pearson correlation coefficients (*r*) and *p*‐values for the correlation of FOXO1 or FOXO3 mRNA expression in various regions of the human brain with the age. (c) The mRNA expressions of FOXO1 and FOXO3 were measured in human cerebellums (*n = *33). Blue and green dots indicate samples used for WB in (d). The mRNA (e) and protein (f) expression of Foxo1, Foxo3, and phospho‐T24/32 Foxo1/3 in young (<3‐month, *n* = 6), adult (3‐18‐month, *n = *6), and old (18–20‐month, *n = *6) FVB/B6 mixed‐strain mouse cerebellums is shown. Each dot represents individual animal. Error bars, mean ± *SEM*. *p < .05; **p < .01; ***p < .005. Statistical significance was determined by unpaired *t*‐test. (g) FOXO1 IHC analysis of brain sections of *WT* and *Foxo 1/3/4 KO* mice. Residual FOXO1 immunoreactivity in *KO* mice is visible in endothelial cells (inset). Scale bar = 200 μm. (h) RT‐qPCR results for Foxo1 and Foxo3 mRNA. Empty bars represent *WT,* and colored bars represent *KO* tissues (*n = *4). (i) Representative Western blotting results. Foxo1‐ or Foxo1/3‐specific knockouts (*1* ^*KO*^ or *1/3* ^*KO*^) selectively lost targeted isoforms. CBM—cerebellum, STR—striatum, SCD—spinal cord, CTX—cortex, BST—brain stem, MDB—midbrain, HPC—hippocampus

To date, there is no clear consensus on the expression of mammalian FOXO in aging tissues despite its evolutionarily conserved role in longevity. We examined publically available datasets of cohorts from studies on aging in the brain for information on FOXO expression (Ramasamy et al., 2014). The expression of FOXO1 significantly increased over the human lifespan in aged (>60 year) brain tissues, while changes in FOXO3 or FOXO4 expressions were less pronounced or not observed at all across different brain regions (Figure 1a,b and Table S1). An independent analysis of FOXO1 and FOXO3 gene expressions in human cerebellums (Figure 1c,d) supported this finding. Expression of FOXO4 or FOXO6 in human cerebellums was low and did not significantly change with age (Fig. S1a). This pattern was also observed in aging mouse brains (Figure 1e,f) and the dataset from a study analyzing multiple mouse strains for age‐associated genes in the cerebellum (Park et al., 2009) (Fig. S1b). We also examined AKT‐dependent phosphorylation of FOXO1/3 (T24/T32) status of mouse age cohort samples. Overall, the levels of phosphorylated FOXO1/3 did not correlate with age (Figure 1f). We measured *HBP1* a bona fide FOXO transcriptional target (Coomans de Brachene et al., 2014) for its mRNA expression as readout of FOXO activity. HBP1 mRNA was increased with aging tightly correlating with FOXO expressions suggesting its activation (Fig. S1c).

From this analysis, we identified 651 genes positively correlating with both increased FOXO1 expression and age across multiple regions of human brain (Table S2). Analysis for transcription factor binding site enrichment within the promoters of these genes identified interferon regulatory factor (IRF, V$IRF3_06, IRF2_Q6, V$IRF_Q6_01) as a potentially important regulator of these genes (395 genes, *p* = 7e‐05). Among the potential IRF‐induced genes was FOXO1 itself with predicted IRF3 binding sites within its proximal promoter sequence. This suggests that FOXO1 expression as well as its coregulated genes in the aging brain may be responding to the inflammatory cytokines that are shown to progressively increase with age in the brain (Bodles & Barger, 2004; Wilson, Finch & Cohen, 2002). Indeed, the stimulation of primary neuronal cultures with cytokines shown to increase in aging brains such as interferons (IFN) or tumor necrosis factor (TNF)‐α (Wilson et al., 2002) induced the expression of both FOXO1 and FOXO3 (Fig. S2a). FOXO increased in both nuclear and cytoplasmic fractions in the presence of cytokines (Fig. S2b,c). Furthermore, the promoter regions of 174 of these genes contained the DAF‐16 family binding element (DBE) FOXO motif, suggesting that they may be direct FOXO targets. Previously reported FOXO targets such as *TXNIP* (Webb, Kundaje & Brunet, 2016) and *HBP1* (Coomans de Brachene et al., 2014) were among these. In contrast to the 651 genes positively correlating with both FOXO1 and age, only 22 genes were found to be negatively correlated with both FOXO1 and age across the same brain regions (Table S2). Transcription factor binding site analysis yielded no significant results on this set, and no genes with DBE motifs were identified. These results show for the first time the coordinated expression of FOXO and its putative targets in the aging mammalian brain as well as the likely dependence of this increased expression on an inflammatory milieu.

### 2.2 Acceleration of aging‐associated axonal tract degeneration in Foxo1/3/4 knockout mice

**Figure 2:**
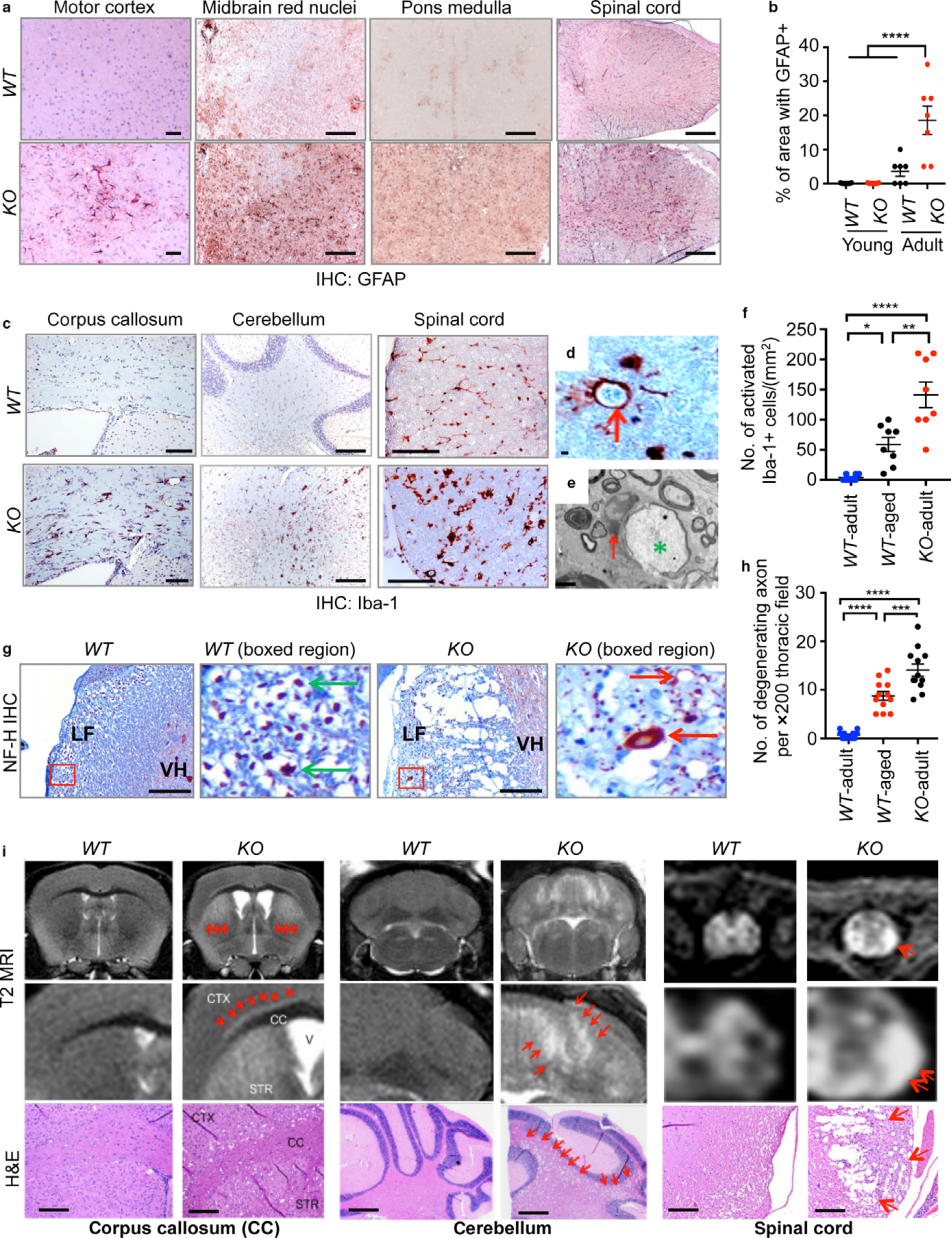
Acceleration of aging‐associated axonal tract degeneration in *Foxo 1/3/4* knockout mice. Axonal tract degeneration and associated reactive gliosis in brains and spinal cords are shown from adult mice as determined by GFAP (a), Iba‐1 (c, d), or Neurofilament H (NF‐H) (g) IHC (*n = *6–8). Quantitation of multiple IHC results is plotted (b, f, h). Error bars, mean ± *SEM*. *p < .05; **p < .01; ***p < .005; ****p < .001. Statistical significance was determined by one‐way ANOVA. (d) Activated phagocytic microglia (red arrow) wraps around cellular debris from adult *KO* spinal cord. (e) Electron microscopy identified microglia (red arrow)‐wrapped axonal remnants (green *). (g) Typical large axons (green arrow) and dystrophic swollen and degenerating axons (red arrow) are shown from boxed areas. LF, lateral funiculus; VH, ventral horn. (i) A close correspondence between cavitations in H&E sections and T2 MRI hyperintensity (red arrows). Scale bars = 200 μm (a, c, g, i) and 2 μm (d, e)

To determine the role of FOXO upregulation in the nervous system during aging, we employed conditional *Foxo* (*Foxo* ^*L*^) alleles and crossed them to human GFAP‐cre, a transgenic mouse line with a nervous system‐specific deletion (Zhuo et al., 2001). Our approach was to delete related FOXO 1, 3, and 4 isoforms given their functional redundancy and their dispensability for neuro‐development (Paik et al., 2007, 2009). We did not include FOXO6 based on its reported role in neocortex development and its low expression outside the hippocampus (Paap et al., 2016; Salih et al., 2012). To better understand the ensuing phenotypes, we first determined the deletion efficiency of FOXO1 and FOXO3, two major isoforms in the brains of wild‐type (*WT*) and *Foxo 1/3/4* combined‐knockout (*KO*) animals. FOXO1 is highly expressed in layer‐V cortical neurons, the Bergman glia of the cerebellum, and the neurons of the caudate putamen and dentate gyrus. This is consistent with the in situ hybridization data from the Allen Brain Atlas (RP_050407_02_B11) and the loss of immunoreactivity observed in *KO* tissues (Figure 1g). Both the mRNA and protein expression patterns (Figure 1h,i) indicate that FOXO3 is expressed throughout the brain agreeing with the previous studies (Paik et al., 2009). Importantly, these animals did not show developmental abnormalities or alterations in brain cytoarchitecture throughout early adulthood (Paik et al., 2009). Instead, an immunohistochemical analysis of astroglia (GFAP^+^) and microglia (Iba‐1^+^) revealed extensive microglial and astroglial activation in the white matter of the brains of adult (10‐ to 15‐month‐old) *KO* mice, which was comparable to that observed in aged (24‐ to 36‐month‐old) *WT* mice (Figure 2a–f and Fig. S3a,b). In mice, rats, rhesus monkeys, and humans, an age‐related increase in white matter astrogliosis and microgliosis has been extensively documented (Bronson, Lipman & Harrison, 1993; Miller & Streit, 2007). Gliosis is likely caused by degenerative changes in the axonal structure (Peters & Rosene, 2003). Indeed, numerous microglia were found surrounding degenerating axons (Figure 2d,e). Importantly, these changes are morphologically indistinguishable from the axonal degeneration evident during aging in aged *WT* animals (Fig. S3c) and occur at a considerably younger age in *KO* mice (Figure 2f). Furthermore, Neurofilament H (NF‐H) staining showed that few intact axons were present with increasing number of degenerating axons in the spinal cord lesion of *KO* mice (Figure 2g,h), Importantly, *Foxo 3/4* double knockout (*3/4* ^*KO*^) animals did not exhibit above‐described axonal degeneration nor reactive microgliosis at advanced age excluding the possibility of cre toxicity and further confirmed functional redundancy among FOXO 1, 3, and 4 isoforms (Fig. S4a,b). Furthermore, in agreement with our histological findings, areas of microglial and astroglial activation in the white matter also presented with extensive cavitation (Figure 2i, H&E); these cavities are most likely the result of degenerating axons and correspond to regions of T2‐MRI hyperintense lesions which typically result from a disorganized ultrastructure. Together, our analysis showed that the effect of aging on the microstructural integrity of the brain in *KO* mice is mainly white matter degeneration, which is the most common and phylogenetically widespread aging‐associated factor in nervous system deterioration (Peters & Rosene, 2003).

### 2.3 A neuron‐specific deletion of Foxo 1/3/4 is sufficient to initiate axonal degeneration

**Figure 3:**
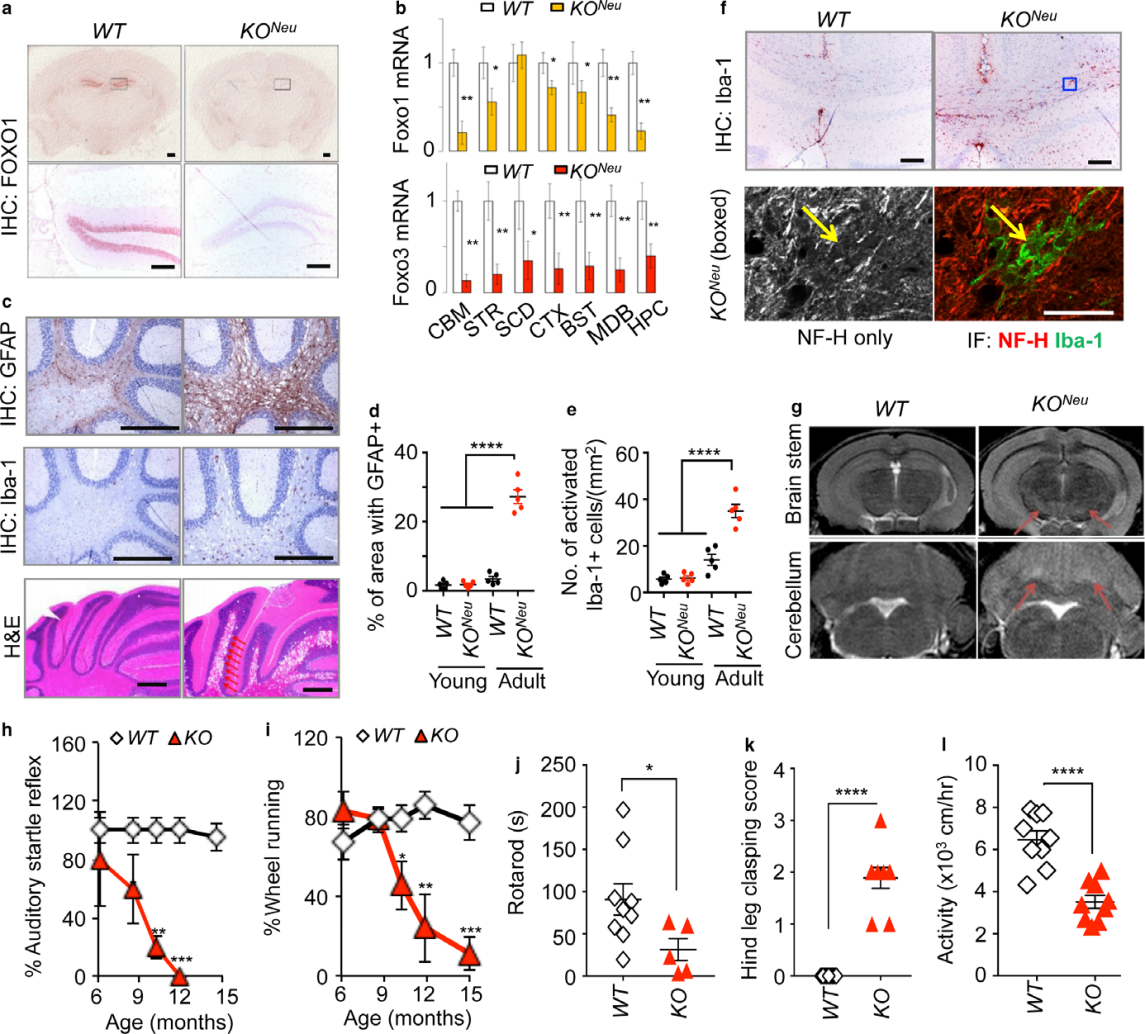
A neuron‐specific deletion of *Foxo 1/3/4* causes axonal degeneration. (a) FOXO1 IHC analysis of brains from mice with the indicated genotypes. Corresponding boxed regions indicate the dentate gyrus and are shown in the lower panels. (b) RT‐qPCR analysis of Foxo1 and Foxo3 mRNA (*n = *4). Colored bars represent *KO*^*N*^ ^*eu*^ tissues. (c) Indicated IHC analysis and H&E staining of cerebellums from adult (15 mo) *KO*^*N*^ ^*eu*^ animals are shown. Arrows point to cavitations caused by degeneration. Scale bar = 200 μm. Quantitation of multiple IHC results is shown for GFAP (d) or Iba‐1 (e). Error bars, mean ± *SEM*. ****p < .001. One‐way ANOVA. (f) Iba‐1 IHC analysis of the hippocampal commissure from adult animals. Blue outline boxed region was further analyzed by co‐IF of NF‐H and Iba‐1 in lower panels. Yellow arrow points to degenerating axon remnant overlapping with microglia. Scale bars = 200 (IHC) and 20 (IF) μm. (g) T2‐MRI of brains in adult mice with the indicated genotypes. Arrows point to hyperintensity lesions. (h) Auditory startle reflexes and (i) the voluntary wheel‐running activity. WT (*n = *7), KO (*n = *5). (j) Rotarod motor coordination test, (k) hind leg clasping score, and (l) locomotor activity of 12‐month‐old *WT* (*n = *9) and *KO* (*n = *5–9). Error bars, mean ± *SEM*. *p < .05; **p < .01; ***p < .005. Statistical significance was determined by unpaired *t*‐test

To investigate the cause of degenerative phenotypes, we determined whether axonal degeneration is due to cell‐intrinsic defects by analyzing *Synapsin 1 (Syn)‐cre+;Foxo1/3/4* ^*L/L*^ (*KO* ^*Neu*^) which exclusively targets mature neurons because *hGFAP‐cre* results in FOXO ablation in both neurons and glial cells due to the activation in common progenitors during early development (Zhuo et al., 2001). In *KO* ^*Neu*^ mice, the efficiency of *Foxo* deletion was overall poor with low penetrance, with highest in neuron‐rich brain regions (Figure 3a,b). Nevertheless, white matter cavitation along with microglial and astroglial activation was evident in the cerebellum of these mice starting at 6 months of age (Figure 3c–e). We determined the axonal tract degeneration of hippocampal commissure where microgliosis was readily visible with Iba‐1 IHC in adult *KO* ^*Neu*^ mice (Figure 3f). Degenerated axon remnants were colocalized with activated microglia in the lesion. These degenerating white matter cavitations were also visible in T2‐MRI in vivo (Figure 3g). From these results, we conclude that the loss of FOXO in neurons is sufficient to initiate aging‐related axonal tract degeneration.

To determine the functional consequence of axonal tract degeneration, we measured motor task performance. We examined *hGFAP‐cre*‐mediated *KO* line because *KO* ^*Neu*^ mice have very low penetrance of cre expression affecting only a small fraction of mature neurons. *KO* mice developed significant motor impairment at advanced ages. Auditory startle reflexes, the voluntary wheel‐running activity, baseline locomotor activity, and the motor coordination dropped precipitously along with increased leg clasping behavior during adulthood in *KO* mice (Figure 3h–l). These results corroborate that FOXO confers and supports physiological health measures via neuroprotection. Together, these results demonstrate that the loss of FOXO accelerates axonal degeneration and ultimately leads to motor dysfunction, a finding consistent with previous study in *C. elegans* (Calixto, Jara & Court, 2012).

### 2.4 Increased proteotoxic stress in Foxo 1/3/4 knockout neurons

To further probe the similarities between the neuropathological changes in *KO* mice and those that occur during natural aging, we compared changes in gene expression between the brains of chronologically aged mice and adult *KO* mice. In a recent study of 12 mouse strains, 63 unique genes were commonly upregulated upon aging in the brain (Park et al., 2009). Of these, *Lzp‐s, Gfap, C1qa, C4B,* and *Ctss* were established as having the most robust changes. All of these genes were strongly induced in the axon‐enriched striatum of adult *KO* mice. This pattern was also found in aged mice relative to adult *WT* mice (Fig. S5a) suggesting that brains lacking FOXO expression share core gene expression changes with chronologically aged ones.

**Figure 4:**
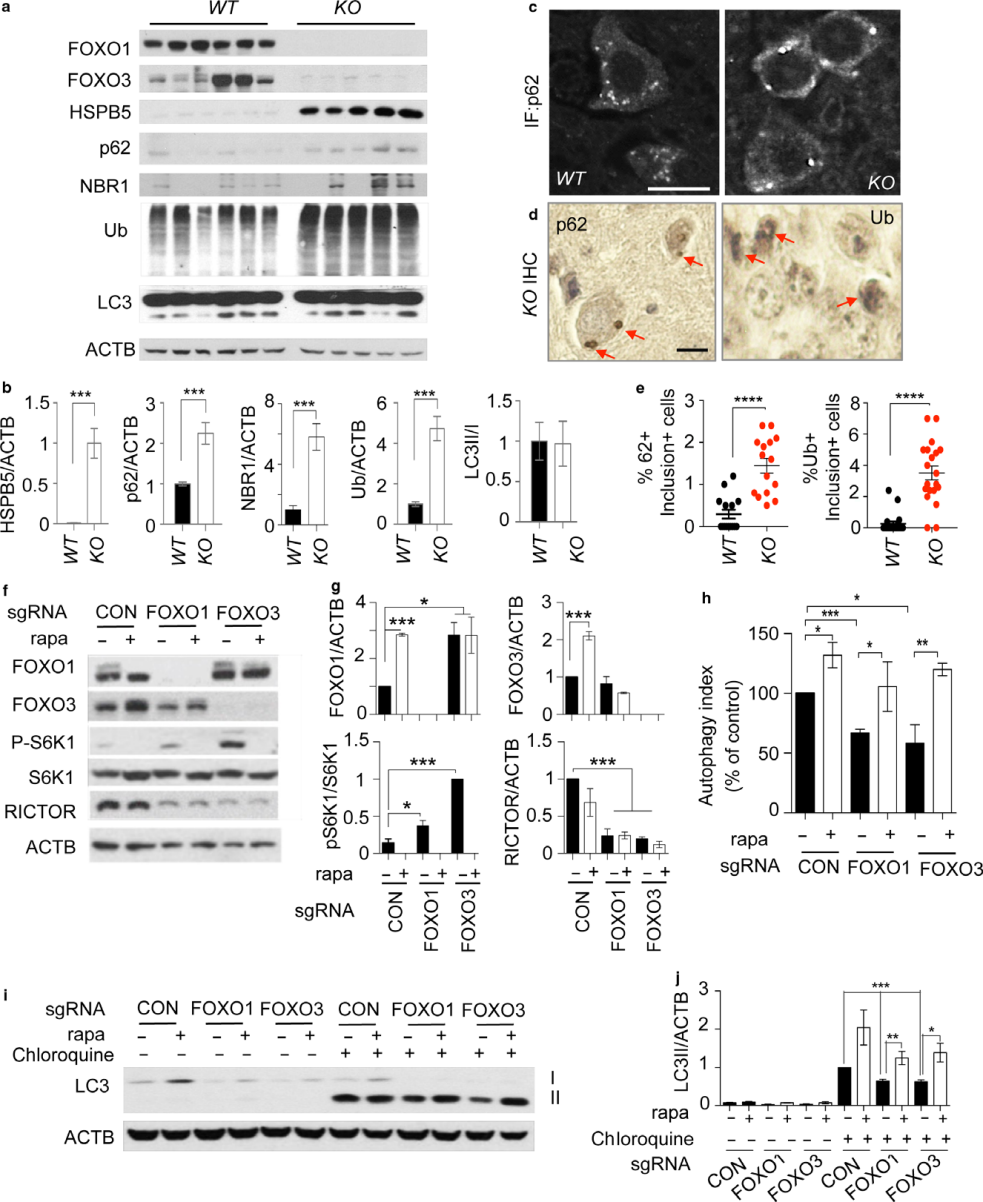
Increased proteotoxic stress and defective autophagy in *Foxo1/3/4* knockout mice. (a) Tissue lysates from 12‐month‐old *WT* and *KO* brain cortex were analyzed by WB of indicated proteins. (c) p62 IF and (d) p62 and Ub IHC on cortical neurons from 12‐month‐old *WT* and *KO* brain cortex. Red arrows point to labeling positive inclusions. Scale bar = 200 μm. (e) Quantitation of representative results for (d). (f) Indicated genotype of neural progenitor cultures was differentiated into neurons for 5 days. Some cultures were treated with rapamycin (100 ng/ml) for 24 hr on day 4 of differentiation. (h) The autophagy activity was analyzed by flow cytometry. The bar graph shows autophagy indices as % of control of (f). (i) Cultures as in (f) were treated with chloroquine (100 ng/ml) for 4 hr and analyzed for the expression levels of LC3 by WB. Representative result from three to five independent experiments is shown. (b, g, j) Quantitation of WB band densities. Only chloroquine‐treated cultures were statistically compared in (j). Error bars, mean ± *SEM*. *p < .05. **p < .01, ***p < .005; ****p < .001. Statistical significance was determined by one‐way ANOVA

We further performed an integrated transcriptomic analysis of laser capture microdissected dentate gyrus neurons to identify the cellular processes that permit this accelerated aging. Dentate gyrus neurons express high levels of FOXO proteins and showed highly efficient deletion in *KO* mice (Figures 1g and 3a). Notably, enriched processes by gene ontology analysis suggested an ongoing elevation of protein aggregation and unfolding in *KO* neurons (Fig. S5b). To validate these results, we analyzed brain cerebral cortex lysates from old *WT* and *KO* animals for the presence of proteotoxic stress. First, we detected significantly upregulated expressions of molecular chaperone *Hspb5* in both mRNA and protein levels in multiple *KO* brain and spinal cord regions (Fig. S6a,b) supporting the role of FOXO in controlling proteostasis in the central nervous system. Furthermore, we found evidences of proteotoxic stress in *KO* brains by measuring the accumulation of ubiquitinated proteins, chaperone SQSTM1 (p62), and a selective autophagy cargo receptor neighbor of BRCA1 (NBR1). p62 and NBR1 bind to the ubiquitin (Ub) and microtubule‐associated protein 1 light chain 3 (LC3) to form protein aggregates: these are degraded by autophagy and their accumulation reflects decreased protein degradation and clearance capacity (Komatsu et al., 2007). *KO* brains showed significantly increased steady‐state levels of p62, NBR1, and ubiquitinated proteins (Figure 4a,b), agreeing with RNA expression profiling analysis suggesting the presence of proteotoxic stress and compromised protein clearance (Fig. S5b). Consistently, p62 and Ub immunolabeling on cortical neurons from adult *WT* and *KO* brain cortex showed accumulation of labeling positive inclusions (Figure 4c–e). In neurons, misfolded or aggregated proteins are cleared by a coordinated chaperone‐mediated autophagy, macroautophagy, or Ub‐proteasome system (Wong & Cuervo, 2010). Ub‐positive inclusions were noted in autophagy‐impaired *Atg5*‐deficient neurons leading to degeneration and motor dysfunction (Hara et al., 2006). We therefore posited defective autophagy might play a causative role in proteotoxic stress of *KO* neurons. However, not unexpectedly, brain cerebral cortex lysates without lysosomal inhibition failed to show consistent differences in LC3‐I processing to LC3‐II form (Figure 4a,b) which is rapidly degraded by lysosomal hydrolysis. Instead, using in vitro‐differentiated neurons with a CRISPR‐mediated knockdown of individual FOXO isoforms, we determined the contribution of FOXO1 and FOXO3 to the regulation of autophagic flux (Figure 4f,g). First, we introduced a dual fluorescent protein‐tagged DsRed‐LC3‐GFP reporter to quantitate autophagy activity (Sheen, Zoncu, Kim & Sabatini, 2011). This reporter allows normalization of autophagy activity measured as the decrease in GFP fluorescence to that in DsRed‐LC3 fluorescence to calculate autophagy index (see experimental procedures). FOXO deficiency profoundly suppressed autophagy flux under basal condition while rapamycin, an inducer of autophagy, treatment partially restored it (Figure 4h). Consistently, both FOXO1 and FOXO3 knockdown cultures showed reduced LC3‐II levels when analyzed after treating cultures with the lysosome inhibitor chloroquine (Figure 4i,j). Collectively, our results support the notion that FOXO is necessary for clearance of ubiquitinated proteins by increasing autophagic flux in neurons.

### 2.5 Increased mTORC1 activity is due to loss of SESN3 expression in Foxo1/3/4 knockout neurons

**Figure 5:**
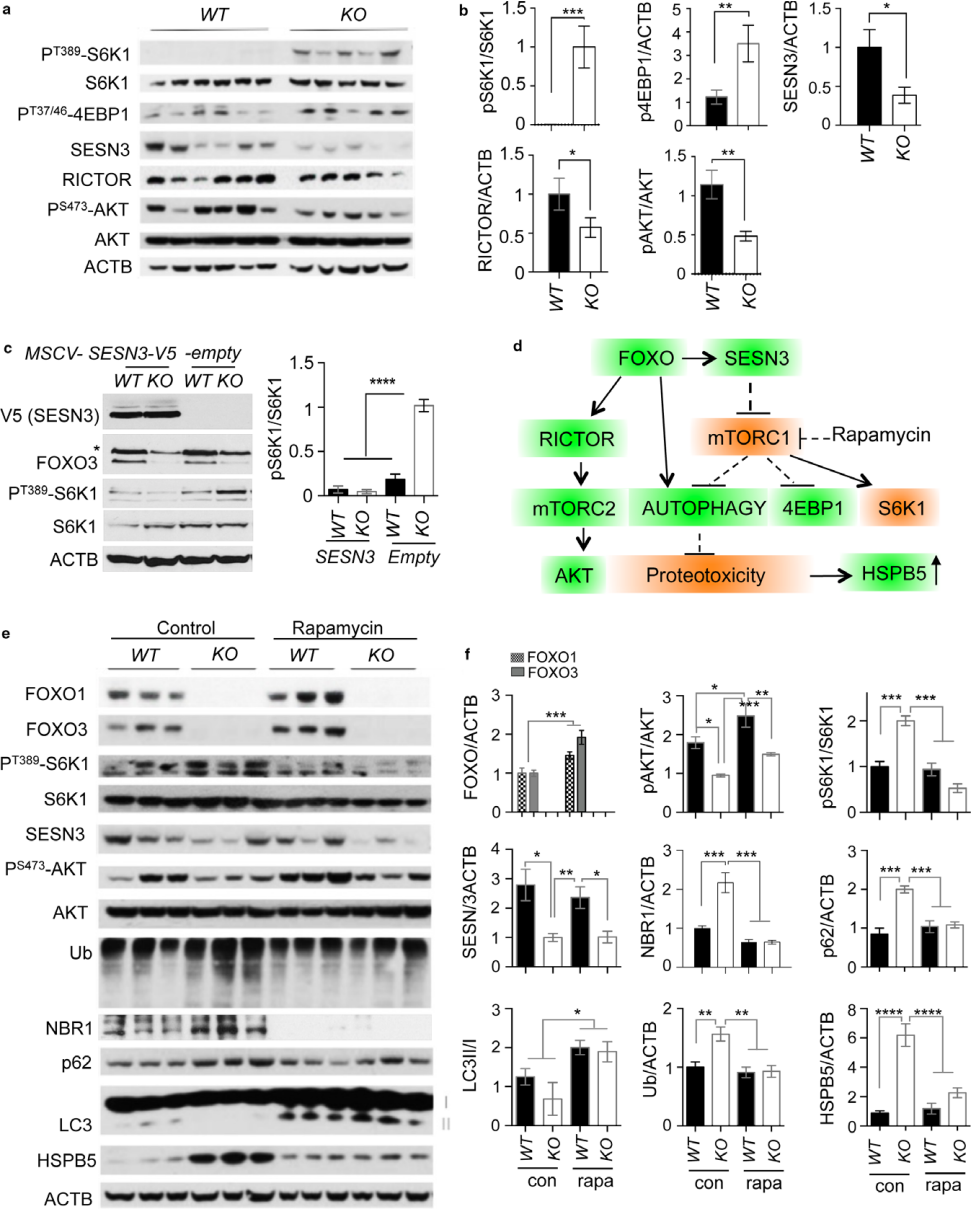
Inhibition of aberrant mTORC1 activation suppresses proteotoxic stress in *Foxo 1/3/4* knockout mice. (a) Tissue lysates from 12‐month‐old *WT* and *KO* brain cortex were analyzed by WB of indicated proteins. (c) Representative WB of primary neuronal cultures from *WT* and *KO* animals infected with indicated retroviral particles. (d) A summary of FOXO and mTORC1 crosstalk leading to the counterbalancing or proteotoxicity. (e) Tissue lysates from 12‐month‐old either rapamycin‐treated or not, *WT* and *KO* brain cortex were analyzed by WB of indicated proteins. (b, c, f) Quantitation of WB band intensities. *p < .05; **p < .01; ***p < .005; ****p < .001. Statistical significance was determined by one‐way ANOVA

We further investigated the mechanistic basis of decreased autophagy in *KO* neurons. Gene expression profiles indicated cellular processes regulating growth and size might be significantly altered (Fig. S5b). We hypothesized that *KO* neurons have hyperactivation of mTORC1 signaling as it is the common regulator of cellular growth as well as autophagy. This then leads to increased cap‐dependent protein translation and imbalanced inhibition of autophagy‐mediated protein degradation. Our findings support this hypothesis: there was a profound elevation of S6K1 (Thr389) and 4EBP1 (Thr37/46) phosphorylation, reflecting activation of mTORC1 in *KO* brains (Figure 5a,b). Mechanistically, our survey of genes in mTORC1 pathway confirmed that SESN3 and RICTOR, bona fide transcriptional targets of FOXO, were significantly downregulated in *KO* brains (Figure 5a,b). Previous in vitro studies also support effector role of SESN3 and RICTOR in FOXO‐regulated mTORC1 and AKT activity, respectively (Chen et al., 2010). Indeed, reduction of SESN3 expression was well correlated with increased phosphorylation of S6K1 and 4EBP1 in *KO* brains (Figure 5a,b). Consistently, RICTOR‐dependent mTORC2 activity was decreased as evidenced by reduced phosphorylation of AKT (Ser473) in *KO* brains (Figure 5a,b). We made similar observations in cultured cells: in vitro‐differentiated neuronal cultures with either FOXO1 or FOXO3 knockdown exhibited elevated mTORC1 activity as shown by increased pS6K1 and reduced RICTOR expression (Figure 4f,g). Furthermore, enforcing the expression of SESN3 in *KO* primary neurons was sufficient to suppress elevated mTORC1 activity as shown by decreased pS6K level (Figure 5c). These results together support the predicted action of FOXO counteracting mTORC1 activity through its transcriptional target SESN3 (Figure 5d).

### 2.6 Inhibition of mTORC1 restores autophagy and prevents axonal degeneration

Next, we determined whether proteotoxic stress and defective autophagy could be corrected by normalizing mTORC1 activity in *KO* brain and spinal cord. We did long‐term (6 months) treatment of rapamycin, a selective mTORC1 inhibitor. First, inhibition of mTORC1 by rapamycin treatment was confirmed by the end point analysis of decreased pS6K1 level. This inhibition appeared direct as SESN3 expression remained significantly low in rapamycin‐treated *KO* brains (Figure 5e,f). The loss of S6K1 activity caused by mTORC1 inhibition disrupts a negative feedback loop, resulting in the reactivation of PI3K–AKT signaling. In agreement, we found that rapamycin induced activation of AKT as evidenced by increased phosphorylation by mTORC2 on Ser473 residue. Notably, the levels of ubiquitinated proteins, p62, and NBR1 in rapamycin‐treated *KO* brains were normalized to the level of *WT* brains indicating enhanced protein degradation and autophagy‐mediated clearance. We noted that LC3‐II levels were largely increased in neuronal cultures and rapamycin‐treated animals suggesting its effectiveness in increasing autophagic flux (Figures 4i,j and 5e,f). At the cellular level, the frequency of cells with large p62 inclusions was decreased and autophagolysosome formation was enhanced as evidenced by increased lysosomal marker LAMP1 and p62 double‐positive punctas in neurons from rapamycin‐treated *KO* animals (Fig. S7).

**Figure 6:**
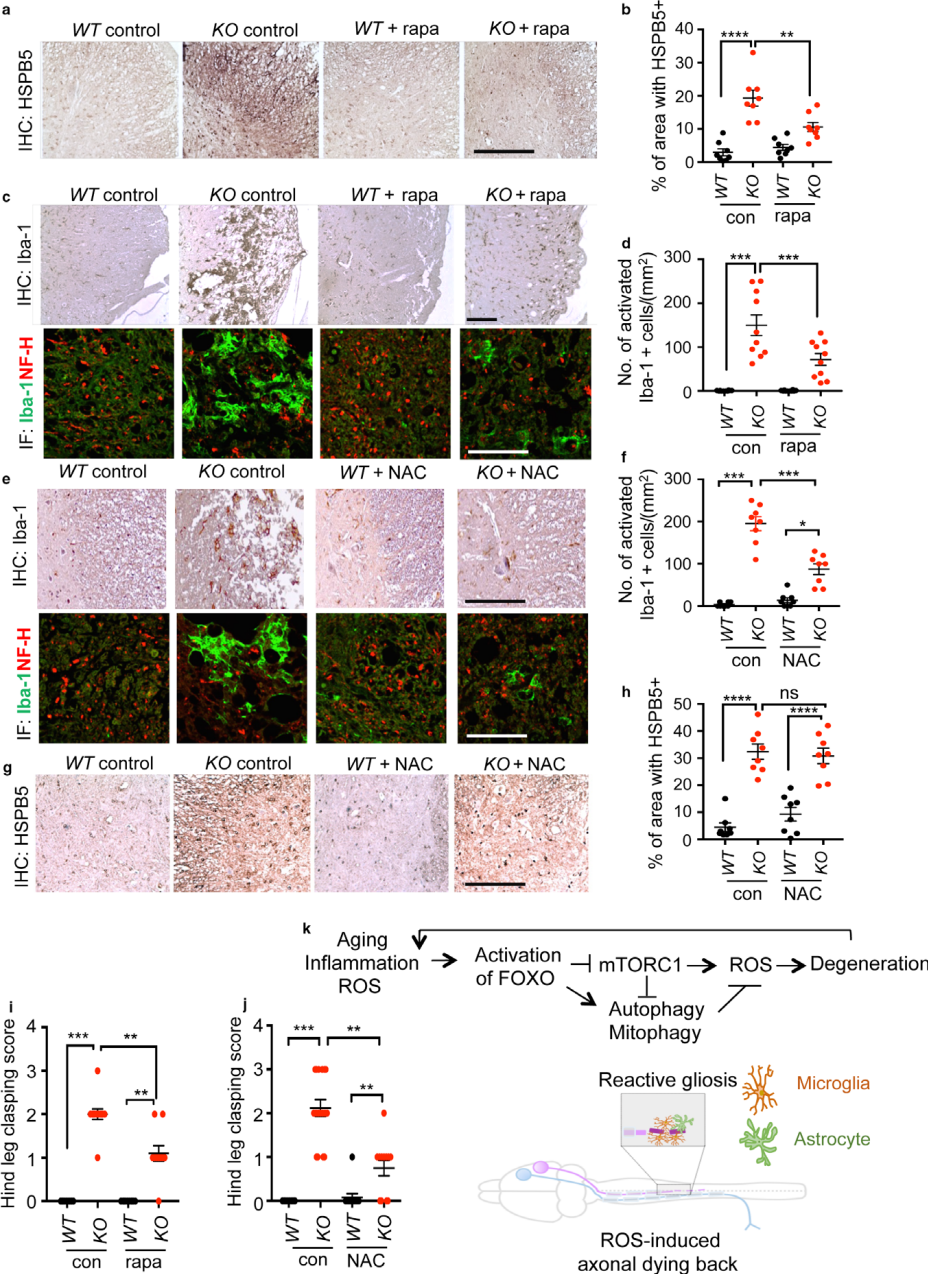
Restoring autophagy and reducing ROS prevent axonal degeneration. HSPB5 (a, g) or Iba‐1 (c, e, upper panels) IHC and NF‐H/Iba‐1 co‐IF (c, e, lower panels) analysis of transverse sections of the thoracic spinal cord (*n = *8–10). Representative lesions in the lateral funiculi from animals treated for 24 weeks from 6 months of age are shown. (b, d, f, h) quantitation of IHC results. Scale bars = 200 μm. Hind leg clasping score of 12‐month‐old *WT* and *KO* mice with or without rapamycin (*n = *10–12) (i) or NAC (*n = *12–17) (j) treatment. Error bars, mean ± *SEM*. *p < .05; **p < .01; ***p < .005; ****p < .001. Statistical significance was determined by one‐way ANOVA. (k) A schematic summary

Our results show that defective clearance of protein inclusions was the cause of increased HSPB5 expression. Rapamycin‐treated mice showed reduced expression of HSPB5 in the brain as well as in the spinal cord (Figures 5e,f and 6a,b). This is consistent with the hypothesis that defective autophagy contributes to the age‐progressive axonal degeneration as the level of Iba‐1‐positive reactive microglia lesions in degenerating lateral thoracic columns was significantly reduced in rapamycin‐treated animals (Figure 6c,d). Furthermore, we determined whether reactive oxygen species (ROS) play a role in driving axonal degeneration as a downstream mediator of defective autophagy. Previously, we reported that FOXO‐deficient neural cells have elevated mitochondria content with increased superoxide suggesting accumulation of defective mitochondria (Yeo et al., 2013). We therefore tested whether the beneficial effect of rapamycin could be explained by restoration of mitophagy and consequential attenuation of ROS generation as previously discussed (Scherz‐Shouval & Elazar, 2011). Antioxidant N‐acetylcysteine (NAC) treatment of presymptomatic *KO* mice suppressed the age‐progressive axonal degeneration and reactive microglia lesions in the spinal cord to a similar degree as rapamycin treatment (Figure 6e,f). Notably, NAC failed to prevent proteotoxicity as determined by HSPB5 expression (Figure 6g,h). These results suggest that ROS is likely to be a downstream mediator of mTORC1 activation‐induced autophagy defect rather than being the cause. In agreement with the improved pathology of the spinal cord, leg clasping behavior reflecting neurodegenerative progression (Guyenet et al., 2010) was prevented by both rapamycin and NAC treatments (Figure 6i,j). Together, our findings support the conclusion that restoration of autophagy capacity prevents age‐progressive axonal degeneration accelerated in *Foxo 1/3/4* knockout mice by suppressing ROS as illustrated in the Figure 6k model.

## 3 DISCUSSION

Our findings reveal a pathway in which pro‐inflammatory signals affect neuronal integrity in the aging brain. Of note, neuroprotective effects of pro‐inflammatory cytokines have been reported while neuroinflammation itself was considered to be the signature pathology of the aging brain. Based on our findings, pro‐inflammatory signals appear to be adaptive and neuroprotective by activating FOXO, but this does not preclude them from being a driver of the aging process. Activation of FOXO may counteract what would otherwise be a trigger for age‐dependent degeneration (i.e., hyperactive mTORC1). In agreement, innate immune responses to tissue damages (*a.k.a*. sterile inflammation) were shown to activate FOXO in an invertebrate model (Obata et al., 2014). It is plausible that axonal degeneration due to loss of proteostasis or FOXO function during aging initiates reactive gliosis to prevent further neuronal damage. This activates pro‐inflammatory signal for the feed‐forward increase of FOXO expression to quell the cause of damage in neurons. Loss of neuronal FOXO function would therefore exacerbate inflammatory responses in aging brain, thus corroborating our findings from the mouse model. Consistently, we observed accumulation of FOXO in the nucleus as well as in the cytoplasm following 48‐h treatment with TNF‐α and IFN‐β in primary neurons (Fig. S2b,c). Collectively, these observations support the notion that timely regulation of FOXO at the level of both expression and activity is necessary for its neuroprotective effect under pro‐inflammatory signals. In line with our model, IFN‐β protects against spontaneous accumulation of protein aggregates in neurons. Lack of IFN‐β signaling led to defects in neuronal autophagy which caused spontaneous neurodegeneration associated with α‐synucleinopathy (Ejlerskov et al., 2015). Whether FOXO plays any roles in cytokine‐induced autophagy warrants further investigation.

Our study demonstrated that depletion of neuronal FOXO 1, 3, and 4 is sufficient to initiate degeneration and to advance aging phenotypes in the brain. However, we do not exclude the possibility that FOXO expression in glial cells is necessary for maintaining white matter integrity in aging animals as its knockout in both neuron and glial cells results in earlier and more profound degenerative phenotypes. Whether the degree of phenotypic severity resulting from employing multiple cre drivers depends solely on different penetrance remains to be determined.

Importantly, we did not observe any apparently degenerating cell bodies or apoptotic cells in the cortical, hippocampal, or cerebellar neurons of *KO* or *WT* mice, even at advanced ages, despite extensive axonal tract degeneration. Also, there was no degeneration in lower motor neurons, grouped atrophy of muscle fibers, or reduction in dopaminergic neurons (not shown), distinguishing age‐progressive axonopathy phenotype from those of other neurodegenerative diseases with different etiologies. Our data agree with the recent paradigm shift in the understanding of brain aging: the loss of cell bodies is considerably rarer than was once thought, and instead, the major aging‐related changes occur in the white matter (Morrison & Hof, 1997; Peters & Rosene, 2003). Furthermore, the mechanism underlying this aging‐related “dying‐back” of distal axons over an extended period has not been clearly understood. In this regard, our study reports FOXO functions to suppress the common neuropathology of chronological aging in mammals: the acceleration of axonal tract degeneration, which is an aging‐related pathology that has been reported in rodents, dogs, and primates (Peters & Rosene, 2003). This may in turn contribute to sarcopenia, peripheral sensory loss, and impaired motor and cognitive function during aging.

Functional and genetic analyses of mTORC1 and FOXO in worms showed both epistatic and parallel interactions in regulating lifespan. Inhibition of mTORC1 and its downstream effector S6K does not require FOXO (Hansen et al., 2007; Pan et al., 2007) while rapamycin‐induced longevity depends on FOXO (Robida‐Stubbs et al., 2012). These studies support the notion that mTORC1 regulates aging by mechanisms that both overlap and distinct from FOXO. Our results that FOXO counteracts mTORC1‐dependent anabolic activity and promotes autophagy activity through its target genes agree with previous studies in other cell types (Webb & Brunet, 2014). Loss of this balance through favoring mTORC1 activation over decreased autophagy by inactivation of FOXO may result in chronic stress and insults to aging neurons. This is highly likely as the inhibition of basal autophagy in the nervous system is sufficient to cause neurodegeneration in mice (Hara et al., 2006). In our experimental model of accelerated brain aging, mTORC1 hyperactivation impairs effective clearance protein aggregates and damaged organelles that contribute to age‐progressive axonal degeneration (Figure 6k). However, it is worth to note that mTORC1 activity may not necessarily increase to contribute to age‐progressive axonopathy. Instead, aging neurons may rely more on autophagy due to decreased neurotrophic factors and impaired nutrient uptake as previously reported in aging hematopoietic cells (Warr et al., 2013). Increased FOXO expression in aging brain may also reflect increased demand on autophagy explaining the beneficial effect of rapamycin. Interestingly, our result also showed that rapamycin upregulates the expression of FOXO proteins in both brain tissue and neuronal cultures (Figure 4f,g and 5e,f). This observation suggests a potential hierarchy of mTORC1 and FOXO regulatory pathways. In addition, mTORC1 inhibition by rapamycin partially restored autophagy in a FOXO knockdown background and suppressed proteotoxic stress in *KO* brain. These results indicate that rapamycin may also work in parallel to or downstream of FOXO action in regulating autophagy. As a result, rapamycin mimics FOXO activation by increasing autophagic flux even in the absence of FOXO.

The upregulation of HSPB5 expression in aging‐related accelerated axonal degeneration in *KO* neurons is mechanistically informative. While a loss of proteostasis and induction of HSPB5 are signature changes of *Foxo*‐deficient neurons, it is notable that NAC treatment partially prevented the degeneration of *KO* animals without improving proteostasis (Figure 6g,h). This observation supports the outweighing role of ROS in driving degeneration. These biochemical changes may represent promising points of intervention in the aging brain. Lastly, the *Foxo* knockout mice may also provide a neurodegenerative disease‐independent model that can be leveraged in the development of therapies to combat age‐related neurodegeneration.

## 4 EXPERIMENTAL PROCEDURES

### 4.1 Generation of experimental mouse cohorts

Nervous system‐specific *Foxo* knockout was generated by crossing floxed *Foxo* mice (Paik et al., 2009) to *hGFAP‐cre* mice. Neuron‐specific *Synapsin1‐cre; Foxo1/3/4* ^*L/L*^ mice in B6 background were kindly provided by Dr. Domenico Accili (Columbia University, NY). Mice were on a mixed background of FVB and B6 if not otherwise indicated. The resulting offsprings were intercrossed to generate mice of the desired genotypes. All animal experiments were approved by Institutional Animal Care and Use Committee. Roughly 1:1 ratio of male and female mice was used for behavioral, imaging, biochemical, and metabolite analyses. For rapamycin or NAC‐feeding experiment, animals were assigned to experimental groups arbitrarily according to age, gender, and genotype without formal randomization. All the animal experiments were repeated multiple times to ensure reproducibility. Animals that show unusual health condition (i.e., runted, rectal prolapse) were excluded from further analysis.

### 4.2 Magnetic resonance imaging

MRI of the brain was performed on a 7 T animal MRI scanner (Bruker, Billerica, MA, USA) with a dedicated mouse head coil. Coronal T2‐weighted images were obtained using the rapid‐acquisition relaxation‐enhanced (RARE) sequence: TR = 3,500 ms, TE = 75 ms, twelve repetitions were acquired and averaged, acquisition time 11 min and 12 s, matrix size 256 × 256, field of view 2.5 × 2.5 cm, slice thickness 0.5 cm, 16 sections acquired. Axial T2‐weighted MRI of the spinal cord was performed using the same RARE sequence except a mouse body coil was used. Representative images from more than 10 animals per genotype analyzed are presented.

### 4.3 Aging brain expression analysis

A gene expression dataset of 134 postmortem human brains dissected into 10 regions was used to assess FOXO1, FOXO3, and FOXO4 expression in relation to subject age at time of death (Ramasamy et al., 2014) (GEO ID: GSE60863). Probe set for FOXO6 was not available. The Affymetrix exon array data were normalized with the Affymetrix RMA sketch algorithm in probe‐level mode and loaded into R2: Genomics Analysis and Visualization Platform (http://r2.amc.nl) for further analysis. For Figure 1a,b, probes 3510861 (FOXO1) or 3748564 (FOXO3) (log2 normalized) were used. The p‐values provided are FDR‐corrected based on an analysis of all genes correlated with age within each brain region. For validation of FOXO expression pattern in aging human brains, 33 cerebellums de‐identified and provided by NIH brain biobank were analyzed by RT‐qPCR as below. Mouse gene expression array data from a published study (Park et al., 2009) were analyzed for FOXO expressions comparing 5‐month vs. 25‐month‐old mice (*n* = 5). For validation, cerebellums from 1.5‐ to 20‐month‐old FVB/B6 mice were analyzed. For gene expression analysis of *WT* vs. *KO* dentate gyrus, laser capture microscopy was performed at Harvard NeuroDiscovery Advanced Tissue Resource Center and analyzed by microarray MOE (430 2.0). CEL files were preprocessed by RMA. The background‐corrected, normalized, and summarized probe set intensity data were then analyzed using significance analysis of microarrays (SAM) to identify differentially expressed genes. Using a twofold, FDR 5% cutoff, we generated a set of 415 probe that distinguishes differentially expressed genes in *WT* vs. *KO*. Differentially expressed gene list was further analyzed by the DAVID Gene Ontology analysis. Dataset GSE102137 is available at (https://www.ncbi.nlm.nih.gov/geo/query/acc.cgi?acc=GSE102137).

### 4.4 Rapamycin or NAC feeding

Both *WT* and *KO* animals at 6 months of age were treated for 24 weeks. LabDiet^®^ 5LG6 with 42 ppm encapsulated rapamycin diets was fed ad libitum. NAC (Sigma) was administered by dissolving in drinking water (40 mM, ad libitum) as previously described (Yeo et al., 2013). Water was replaced weekly.

### 4.5 Assessment of sensorimotor function

We determined the time of paresis onset and the rate of progression for all animals by biweekly assessment of hindlimb clasping according to Guyenet, et al. (Guyenet et al., 2010). Briefly, if the hindlimbs are splayed outward, away from the midline, it is assigned a score of 0. If one hindlimb is retracted to the abdomen for more than 50% of the time suspended, it receives a score of 1. If both hindlimbs are partially retracted toward the abdomen for more than 50% of the time suspended, it receives a score of 2. If its hindlimbs are entirely retracted and touching the abdomen for more than 50% of the time suspended, it receives a score of 3. The paresis progresses to full paralysis, ultimately necessitating humane sacrifice. The age of IACUC‐mandated sacrifice of mice was plotted as paresis‐free survival curve. For motor coordination and balance, we used the rotarod performance test. Subjects were given three training trials to learn to walk on a rotating beam prior to the actual test. Rotarod treadmill (version 8, IITC Lifescience, Woodland Hills, CA) was used with 4–45 rpm acceleration over 2 min. For acoustic startle reflex test, mice were placed in an SR‐pilot startle reflex apparatus (San Diego Instruments, San Diego, CA). Background noise was 65 dB. After a 5‐min habituation period, startle stimuli (115 dB) were presented and responses were recorded. For voluntary wheel‐running activity, total distance from individually housed mice over a week period was recorded. Locomotor activity was evaluated in an open field (45 × 45 cm). The total distance moved was recorded every 10 min for 120 min using a video tracking system (Ethovision 3.0, Noldus Technology). Neurological scoring and motor function measurements were performed by an independent researcher blinded to the experimental conditions.

### 4.6 Immunohistochemistry (IHC) and immunofluorescence (IF)

Formalin‐fixed, paraffin‐embedded 5‐micrometer sections were used. For stains of brain, sections were processed in standard method and incubated with primary antibodies to rabbit anti‐FOXO1 (1:100, #2880, Cell signaling technology) and rabbit anti‐Iba1 (1:500, #019‐19741,Wako), rabbit anti‐GFAP (1:400, #Z0334, DAKO), mouse anti‐Neurofilament H (1:1,000, clone SMI32, Millipore), mouse anti‐HSPB5 (10 μg/ml, Clone1B6.1‐3G4, Stressgen), mouse anti‐p62 (1 μg/ml, #56416, Abcam), rat anti‐LAMP1 (10 μg/ml, #25245), mouse anti‐Ubiquitin (1: 300, #3936, Cell signaling technology) antibodies and further processed by Vector Elite ABC peroxidase kit followed by developing with DAB or NovaRED substrate and counterstained with hematoxylin. Transverse sections of the spinal cord were treated with a permeabilization/blocking solution containing 10% FCS, 2% bovine serum albumin, 1% glycine, and 0.05% Triton X‐100. Primary antibodies were diluted in PBS containing 0.05% Triton X‐100, 0.1% Tween‐20, and 2% horse serum and applied for 1 hr in a humidified chamber at room temperature. For IF of FOXO in cultured primary neurons, cells were fixed in 3.7% paraformaldehyde and permeabilized with 0.2% Triton X‐100. Primary antibodies were added in 3% BSA/PBS and incubated overnight at 4 degrees followed by Alexa Fluor 488‐conjugated donkey anti‐rabbit (1:1,000; #A21206, Thermo Fisher) secondary antibody labeling.

### 4.7 RT‐qPCR analysis

RNA was harvested using GeneJET RNA purification kit (Fermentas) and treated with RQ1 RNase‐free DNase (Promega). Then, one microgram of total RNA was reverse‐transcribed into cDNA utilizing Maxima^®^ first‐strand cDNA synthesis kit for RT‐qPCR (Fermentas). PCR was performed on cDNA samples using the Power SYBR^®^ Green Master mix (Applied Biosystems) and was performed the PCR on the StepOnePlusTM Real Time PCR System (Applied Biosystems). Primer sequences are as below. Each sample was run as duplicates (triplicates), and the mRNA level of each sample was normalized to that of the 18s mRNA. The relative mRNA level was presented as unit values of 2^[Ct(18s) – Ct(gene of interest)]^. Primers are as follows:

**Table:** 

h18s	F	CCGATAACGAACGAGACTCTGG
	R	TAGGGTAGGCACACGCTGAGCC
hFOXO1	F	CAAGAGCGTGCCCTACTTCAA
	R	CAGCTCGGCTTCGGCTCTTA
hFOXO3	F	GTGCGTTGCGTGCCCTACTTC
	R	CATTCTGGACCCGCATGAATCG
hFOXO4	F	CAGGCCATTGAAAGCGCCC
	R	GCCTCGTTGTGAACCTTGATGA
hFOXO6	F	CGCAGATCTACGACTGGATGGT
	R	CACCACGAACTCTTGCCGGT
m18s	F	CCGATAACGAACGAGACTCTGG
	R	AGGGTAGGCACACGCTGAGCC
mFOXO1	F	CTACGAGTGGATGGTGAAGAGC
	R	CCAGTTCCTTCATTCTGCACTCG
mFOXO3	F	CCTACTTCAAGGATAAGGGCGAC
	R	GCCTTCATTCTGAACGCGCATG
mHBP1	F	CCTCTCCAGGATACAACTCCTGTGA
	R	GGTATATGGCAGATTGGGTAGGGT

### 4.8 Western blot analysis

Cells or tissues were directly lysed in Laemli SDS sample buffer, and protein concentration was determined by DC Protein Assay Reagents (Bio‐Rad). Immunoblots were performed with the following antibodies: FOXO1 (#2880, 1:1,000), phospho‐FOXO1 (Thr24)/FOXO3 (Thr32) (#9464, 1:1,000), phospho‐S6 (#4858, 1:2,000), S6 (#2317, 1:2,000), phospho‐S6K1 (#9234, 1:1,000), S6K1 (#2708, 1:2,000), phosphor‐AKT (#4060, 1:2,000), AKT (#9272, 1:5,000), phospho‐4EBP1 (#2855, 1:1,000), 4EBP1 (#9644, 1:2,000), Ubiquitin (#3936, 1:3,000), Rictor (#2114,1:1,000), LC3A/B (#12741, 1:3,000), NBR1 (#9891, 1:1,000), HSPB5 (#8851, 1:1,000) from Cell Signaling Technology, FOXO3 (#NB100‐614, 1:2,000, Novus), β‐actin (#A5316, 1:5,000, Sigma), p62 (#ab65416, 1:5,000, Abcam), V5 (# ab27671, 1:5,000), and Sestrin3 (#H00143686‐M02, 1:1,000, Abnova).

### 4.9 Autophagy analysis in cultures

To measure autophagic activity by flow cytometric analysis, we used a dual‐color autophagy reporter DsRed‐LC3‐GFP (Sheen et al., 2011). pQCXI Puro DsRed‐LC3‐GFP (Addgene # 31182) was used to generate retroviral particles to infect mouse neural progenitors. Cells were subsequently differentiated in B27 containing N2 media for 5 days prior to the analysis. The autophagy index was calculated with the formula: 100 – (100 × (FL1/FL2)) for measuring relative change in GFP to DsRed. The FL1 is the median fluorescence intensity of GFP fluorescence, and FL2 is the median fluorescence intensity of DsRed fluorescence. FOXO knockdown mouse neural progenitors were generated by infecting immortalized *Ink/Arf* null postnatal mouse neural progenitors with lenti‐CRISPR vectors with a specific sgRNA: FOXO1, CGACAGCGGCCCGGTCGGTG; FOXO3, GCGGGTGATCAGGTCGGCAT; CON (Scr), GCACTACCAGAGCTAACTCA or Addgene # 51764.

### 4.10 Statistical analysis

We determined experimental sample sizes on the basis of preliminary data. The size of experimental groups and statistical test applied are indicated in the figure legends. All results are expressed as mean ± *SEM*. GraphPad Prism software (version 7, San Diego, CA) was used for all statistical analysis. Normal distribution of the sample sets was determined before applying unpaired Student's two‐tailed *t*‐test for two group comparisons. One‐way ANOVA was used to assess the differences between multiple groups. The mean values of each group were compared by the Bonferroni's post hoc procedure. Differences were considered significant when *p* < .05.

## AUTHOR CONTRIBUTIONS

I.H, H.O, E.S, D.K, J.W.L, F.L.M, R.T.B, W.H.Y, M.T, and J.P. designed experiments, analyzed data, and wrote the manuscript. I.H, H.O, D.K, J.W.C, J. L., S.R., Y.N., F.L.M, and J.P. performed experiments. E.S. and F.L.M. performed bioinformatic analysis. I.H. and H.O. contributed equally.

## CONFLICT OF INTEREST

The authors declare no competing financial interest.
