# Supplementary material for: FOXO protects against age‐progressive axonal degeneration
Source: Aging Cell. 2017 Nov 26;17(1):e12701. doi: 10.1111/acel.12701 (PMC5771393; doi:10.1111/acel.12701)
Supplement: Supplementary file 1 [file ACEL-17-na-s001.pdf]

Supplemental Information (2 tables and 7 Figures)

FOXO protects against age-progressive axonal degeneration

Supplemental Figure Legend

Supplemental Table S1. *r* values for the correlation of each probe with age for FOXO4 in all 10 brain regions.

Supplemental Table S2. List of genes based on their correlation with FOXO1 and age in all regions of the brain. The Age-Correlation table lists all the *r* and p-values for the individual brain regions for the correlation of these genes with age. The FOXO1-Correlation table lists the values for the correlation of these genes with FOXO1 expression. There are 1431 probes in the table corresponding to 651 genes for positively correlated and 49 probes corresponding to 22 genes for negatively correlated ones.

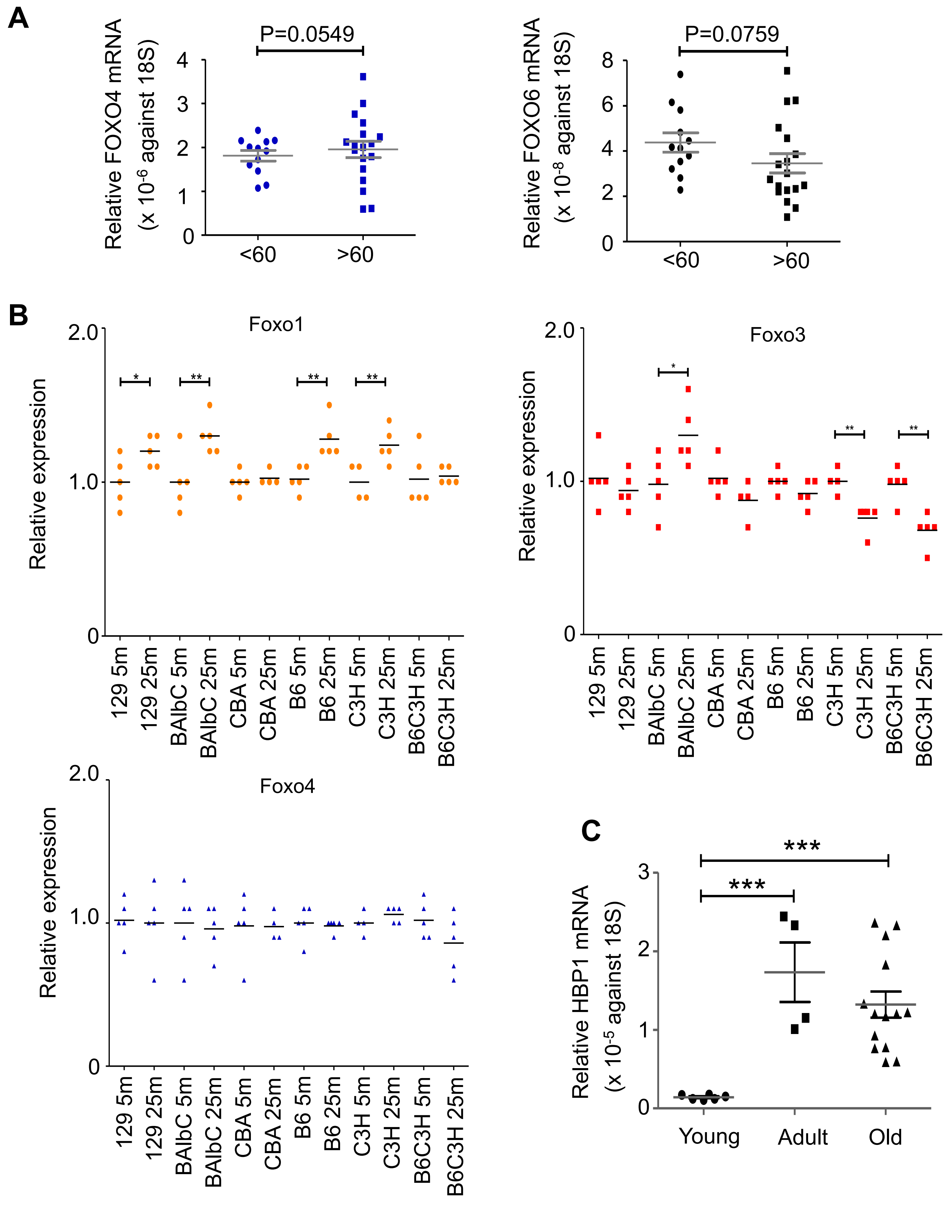

Figure S1. Expression of FOXO in aging brains. (A) Relative mRNA expressions of FOXO4 and FOXO6 in human CB in two age groups as in Fig. 1c. (B) Relative mRNA expressions of *Foxo1*, *Foxo3*, and *Foxo4* in indicated mouse strains. Fold differences are expressed as value of aged (25 month-old) relative to early adult (5 month-old) mice (n=5). (C) Relative mRNA expressions of HBP1 in CB from indicated age groups of mice. Error bars, mean ± s.e.m. *P < 0.05; **P < 0.01; ***P < 0.001. Statistical significance was determined by unpaired t-test.

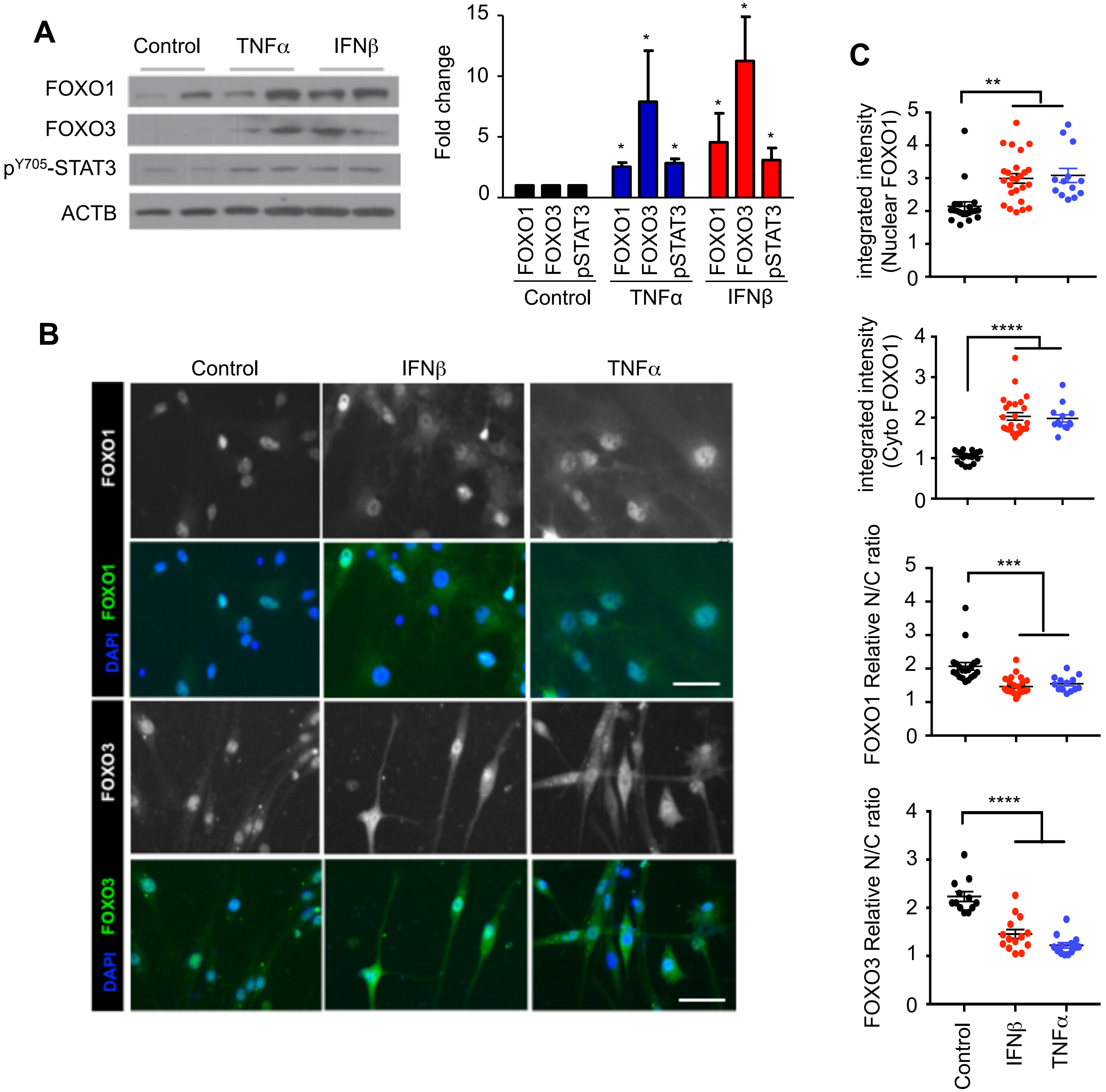

Figure S2. Expression of FOXO under pro-inflammatory stimulation in primary neuronal cultures. Primary mouse neuronal cultures were treated with TNFα (10 ng/ml) or IFNβ (100 ng/ml) for 48 h and analyzed for the expression levels or subcellular localization of the indicated proteins by WB (A) and IF (B-C). Representative result from 3 independent experiments is shown. Densitometry analysis of protein bands is plotted on the right. (C) Relative ratio of **N**uclear to **C**ytoplasmic localization of FOXO1 and FOXO3 is plotted for cultures treated as indicated. Error bars, mean ± s.e.m. *P<0.05, **P<0.01, ***P <0.005, ****P < 0.001. Statistical significance was determined by one-way ANOVA. Bar = 50 μm

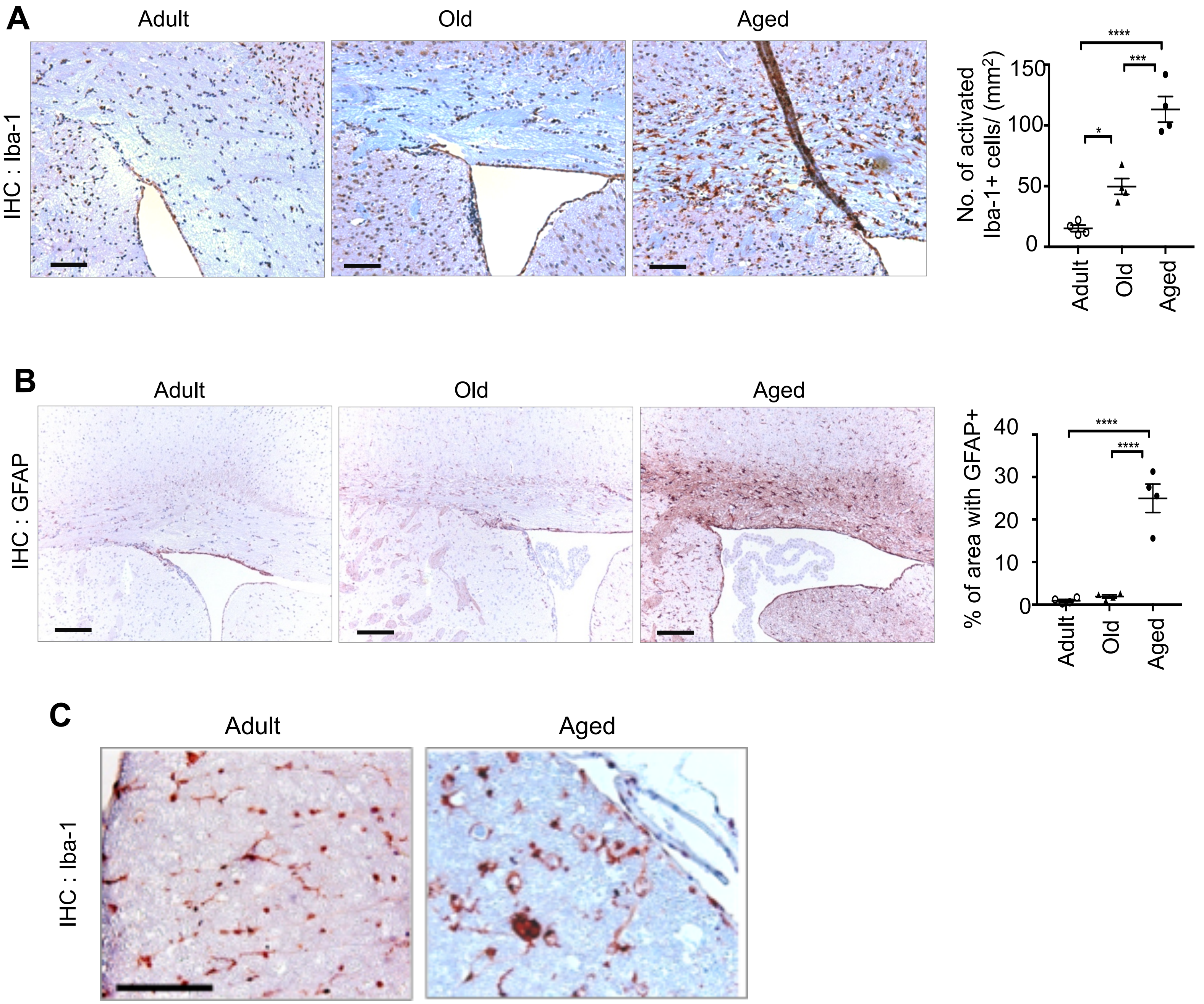

Figure S3. Reactive gliosis and microgliosis increase during chronological aging in mice. Age-progressive increase in reactive gliosis is shown by the activated Iba-1-positive microglia (A) and by the increase in GFAP-positive astrocytes (B) in the corpus callosum above the ventricles and (C) the spinal cord. Shown are adult (10 months), old (22 months), and aged (36 months) representative animals. Quantitation of multiple IHC results is plotted on the right. Error bars, mean ± s.e.m. *$P < 0.05$; ***$P < 0.005$; ****$P < 0.001$. Statistical significance was determined by one-way ANOVA. Scale bar = 200 μm

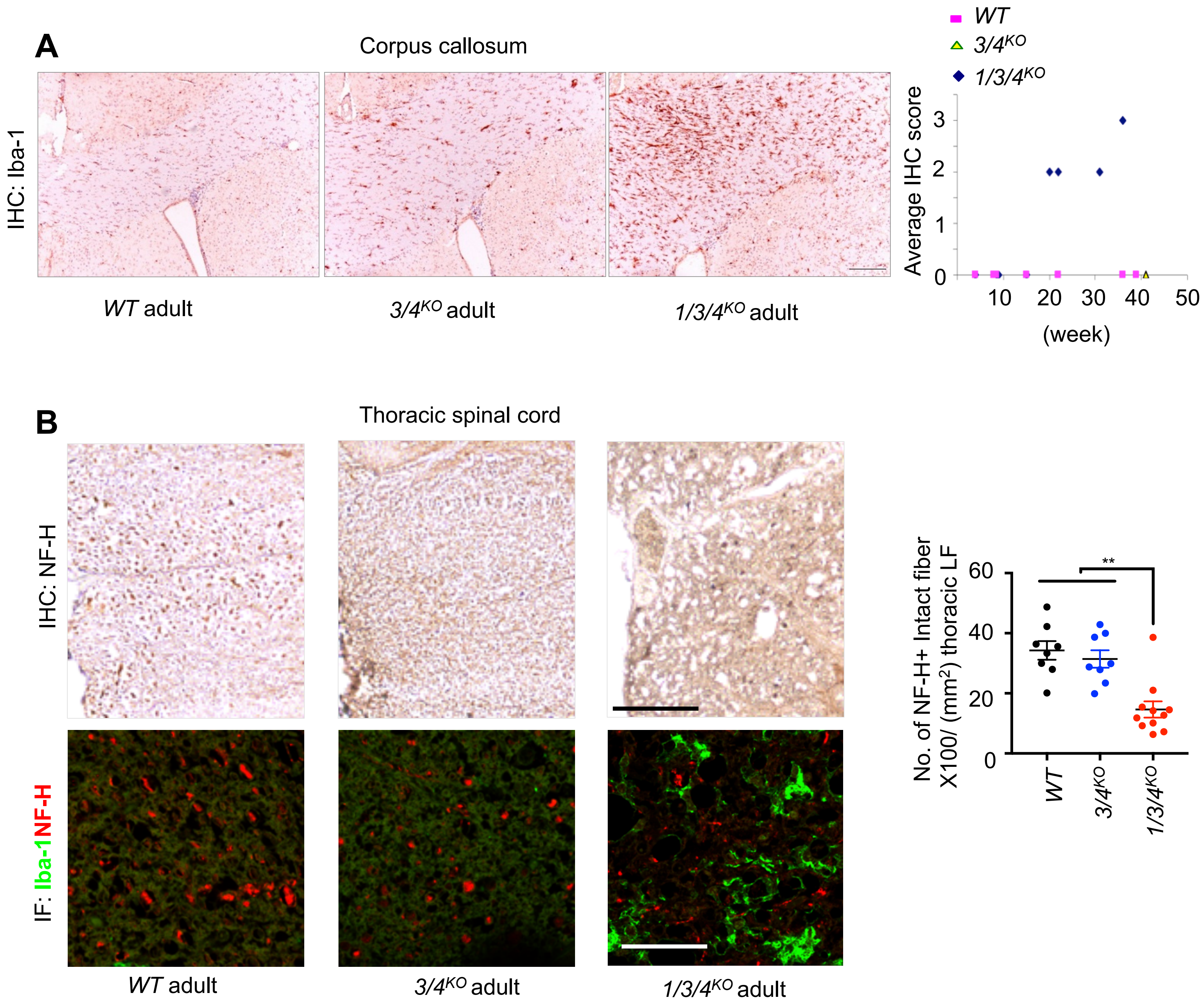

Figure S4. Aging-related axonal degeneration and gliosis pathology are independent of cre expression. (A) Representative image of reactive microgliosis in 15 month-old *WT (*no cre), *3/4^{KO}* (hGFAP-cre+), and *1/3/4^{KO}* (hGFAP-cre+) mice as determined based Iba-1 IHC. Scale bar= 200 μm. Right, a plot of the qualitative score of microgliosis from the corpus callosum of animals of the indicated ages and genotypes (n=6 per genotype). Samples were assigned a score of 0 (no staining), 1 (<10% of positive), 2 (10%-50% of cells positive), or 3 (>50% of cells staining positive) within the microscopic field. (B) The lesion in *1/3/4^{KO}* thoracic spinal cord is absent in 15 month-old *WT* or *3/4^{KO}* . Shown are thoracic lateral funiculus of indicated genotype animals stained for NF-H (IHC) or Iba-1 with NF-H (co-IF). Right, NF-H staining positive axons were scored. Degenerated axonal remnants associated with Iba-1 staining were excluded from scoring. Scale bars = 100 μm (IHC), 50 μm (IF).

A

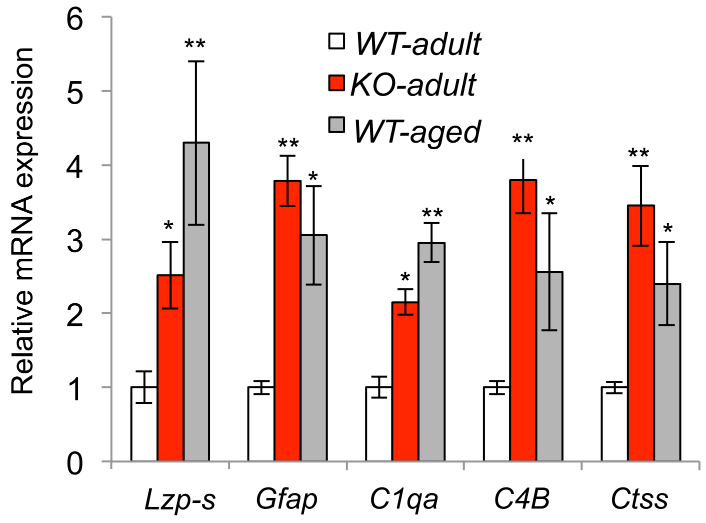
Relative mRNA expression
6
5
4
3
2
1
0
WT-adult
KO-adult
WT-aged
Lzp-s
Gfap
C1qa
C4B
Ctss

B

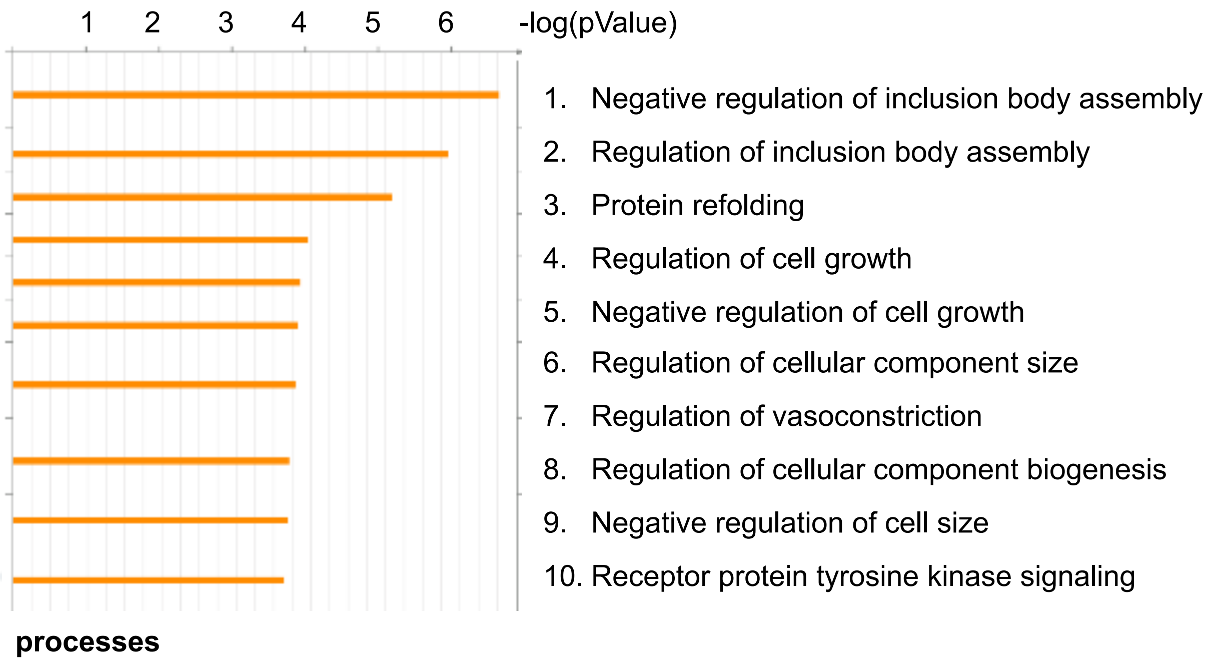
1
2
3
4
5
6
-log(pValue)
1. Negative regulation of inclusion body assembly
2. Regulation of inclusion body assembly
3. Protein refolding
4. Regulation of cell growth
5. Negative regulation of cell growth
6. Regulation of cellular component size
7. Regulation of vasoconstriction
8. Regulation of cellular component biogenesis
9. Negative regulation of cell size
10. Receptor protein tyrosine kinase signaling
processes

B (continues)

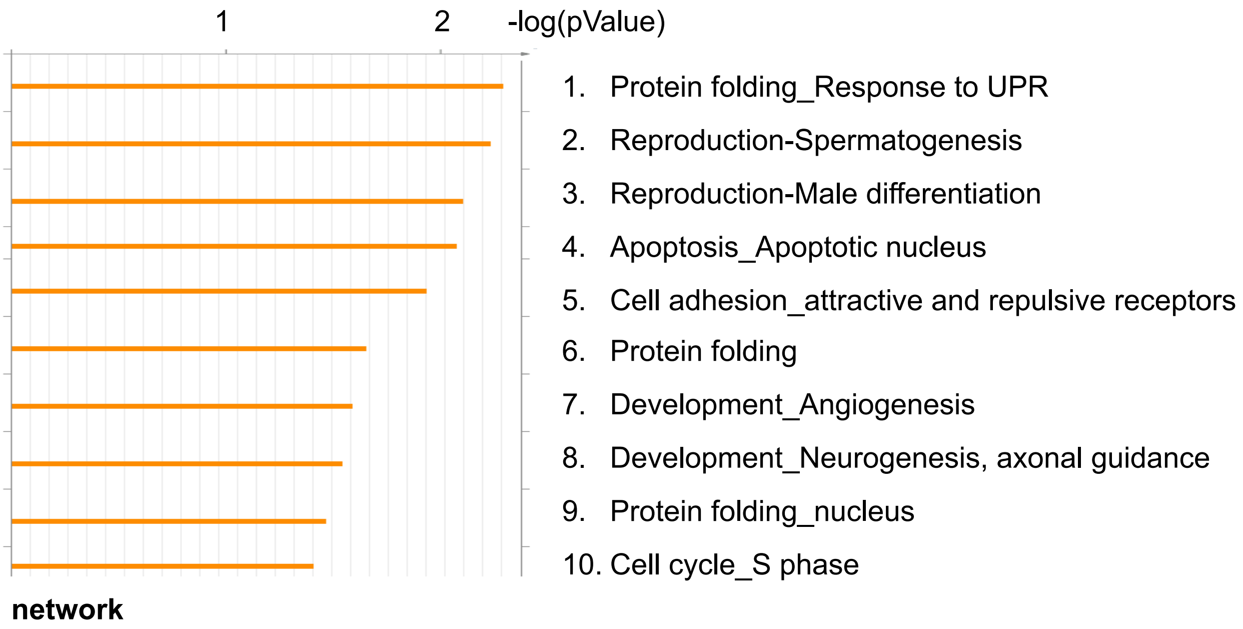

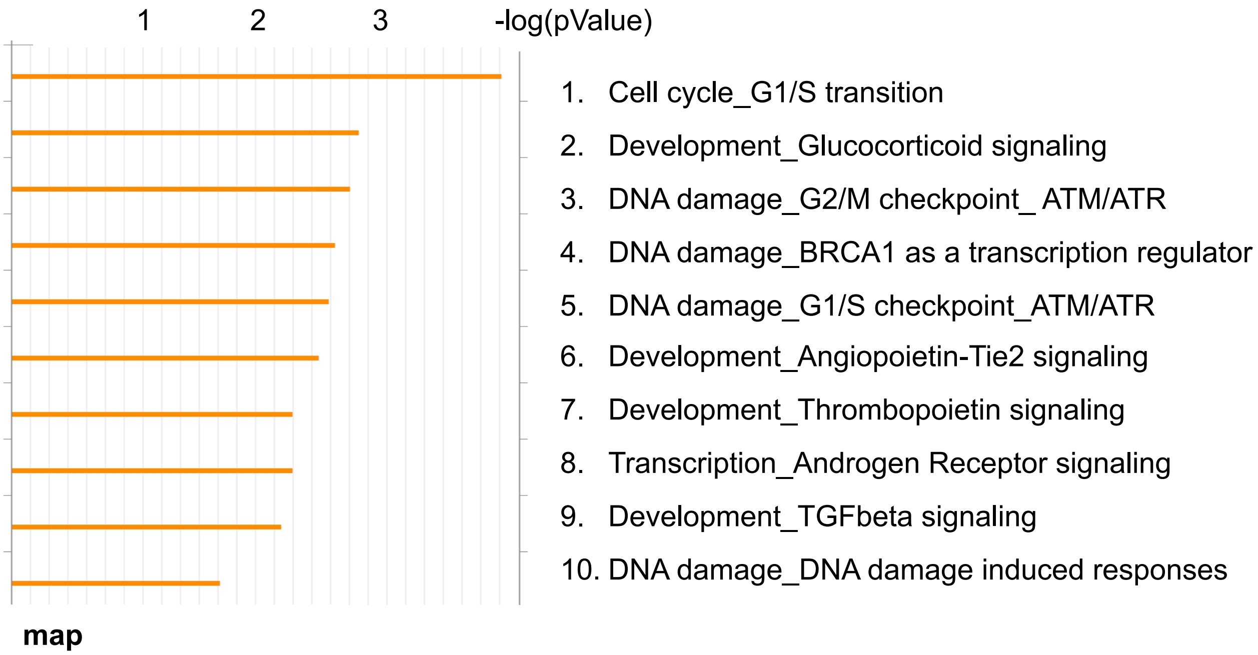

Figure S5. Expression of signature aging genes is increased in *KO* mice. (A) The expression of *Lzp-s, Gfap, C1qa, C4B,* and *Ctss*, which constitute a conserved expression signature of aging in the mouse brain, were measured via qRT-PCR in the striatum of adult (10-12-month old) *WT* (white bars), *KO* (red bars) mice, as well as in aged (25-36-month old) *WT* mice (grey bars). Each Bar shows the average for 3 to 4 animals. Error bars, mean ± s.e.m. $*P < 0.05$; $**P < 0.01$. Statistical significance was determined by one way ANOVA. (B) DAVID Gene Ontology analysis of differentially expressed genes of old *KO* vs *WT* dentate gyrus. Significantly enriched cellular processes, networks, and maps are listed.

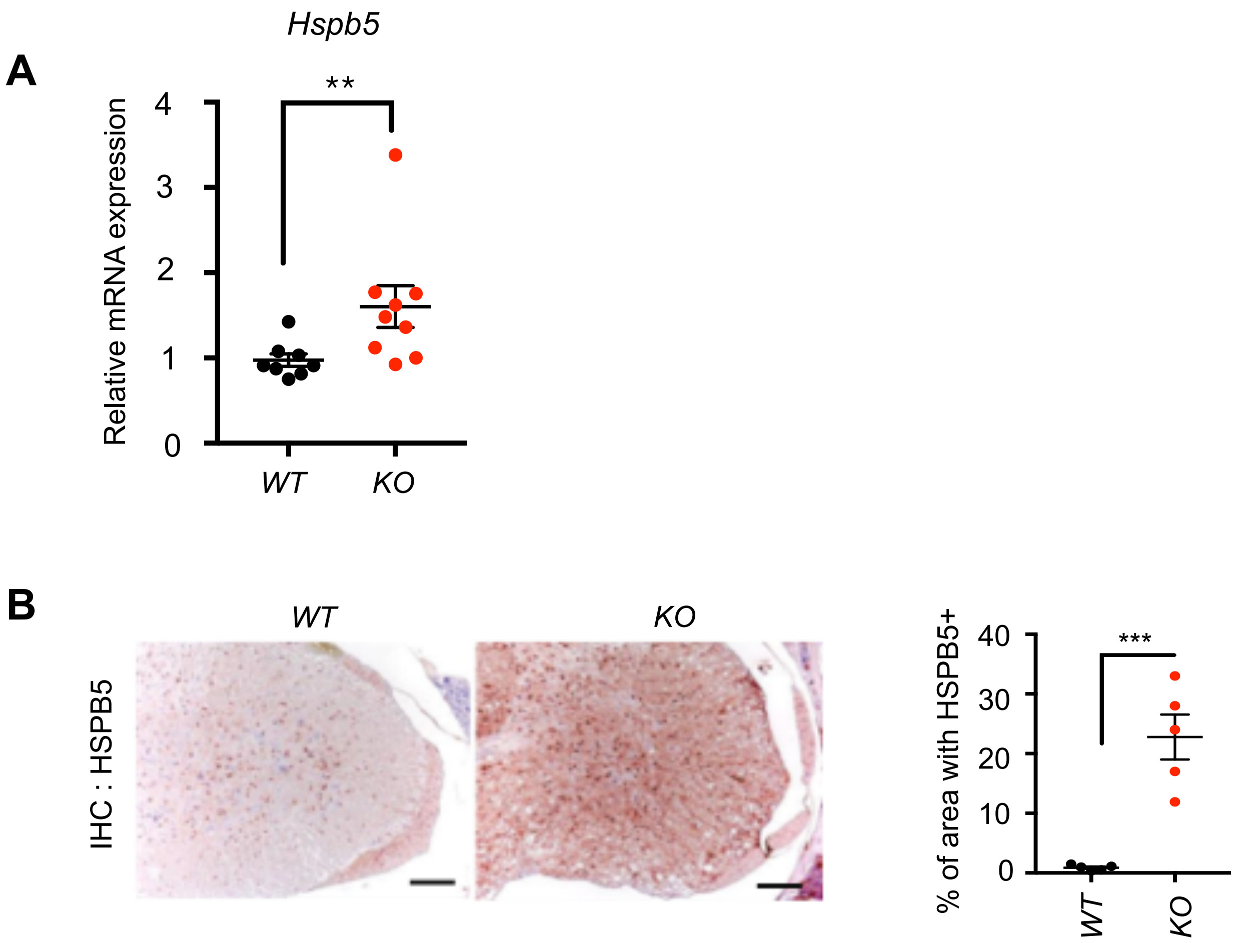

Figure S6. Expression of HSPB5 is increased in *KO* mice. (A) Relative expression of Hspb5 in mouse hippocampi measured by RT-qPCR. *WT* (n=9) and *KO* (n=8) adult mice (13-15 months old) were analyzed. Error bars, mean ± s.e.m. *P < 0.05. Student unpaired t-test was used. (B) The increased expression of HSPB5 was confirmed by IHC in the spinal cord of *KO* mice. Quantitation of multiple IHC results is plotted on the right. Error bars, mean ± s.e.m. ***P < 0.005. Student unpaired t-test was used. Representative results are shown. Scale bars = 200 μm

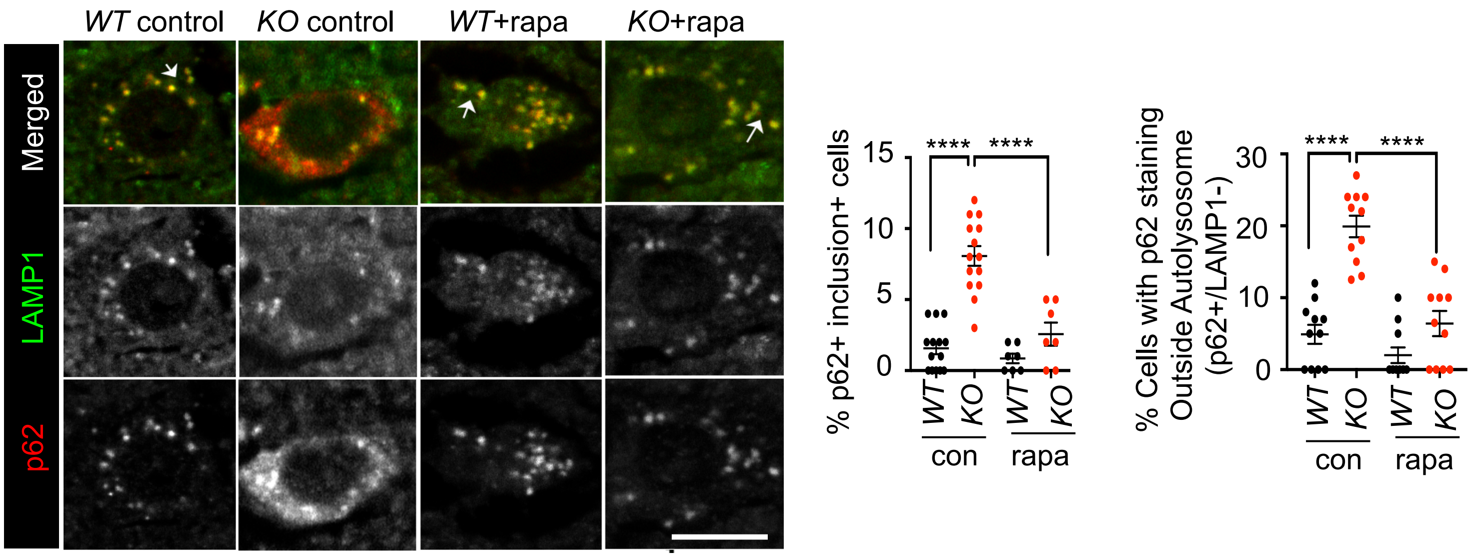

Figure S7. p62 positive inclusions increased in *KO* neurons in an mTORC1 dependent manner. Left, Co-IF for p62 and lysosomal marker LAMP1 in neurons from transverse sections of the thoracic spinal cord. Scale bar = 10 µm. Right, quantitation of IF images for p62 positive inclusions (left). Cells with p62 staining non-overlapping with LAMP1 were scored (right). Error bars, mean ± s.e.m. *P < 0.05; **P < 0.01; ***P<0.005; ****P < 0.001. Statistical significance was determined by one-way ANOVA.
